# Supplementary material for: Hyperoxidized Species of Heme Have a Potent Capacity to Induce Autoreactivity of Human IgG Antibodies
Source: Int J Mol Sci. 2023 Feb 8;24(4):3416. doi: 10.3390/ijms24043416 (PMC9960230; doi:10.3390/ijms24043416)
Supplement: Supplementary file 1 [file ijms-24-03416-s001.zip › ijms-2164251-supplementary.pptx]

## Slide 1
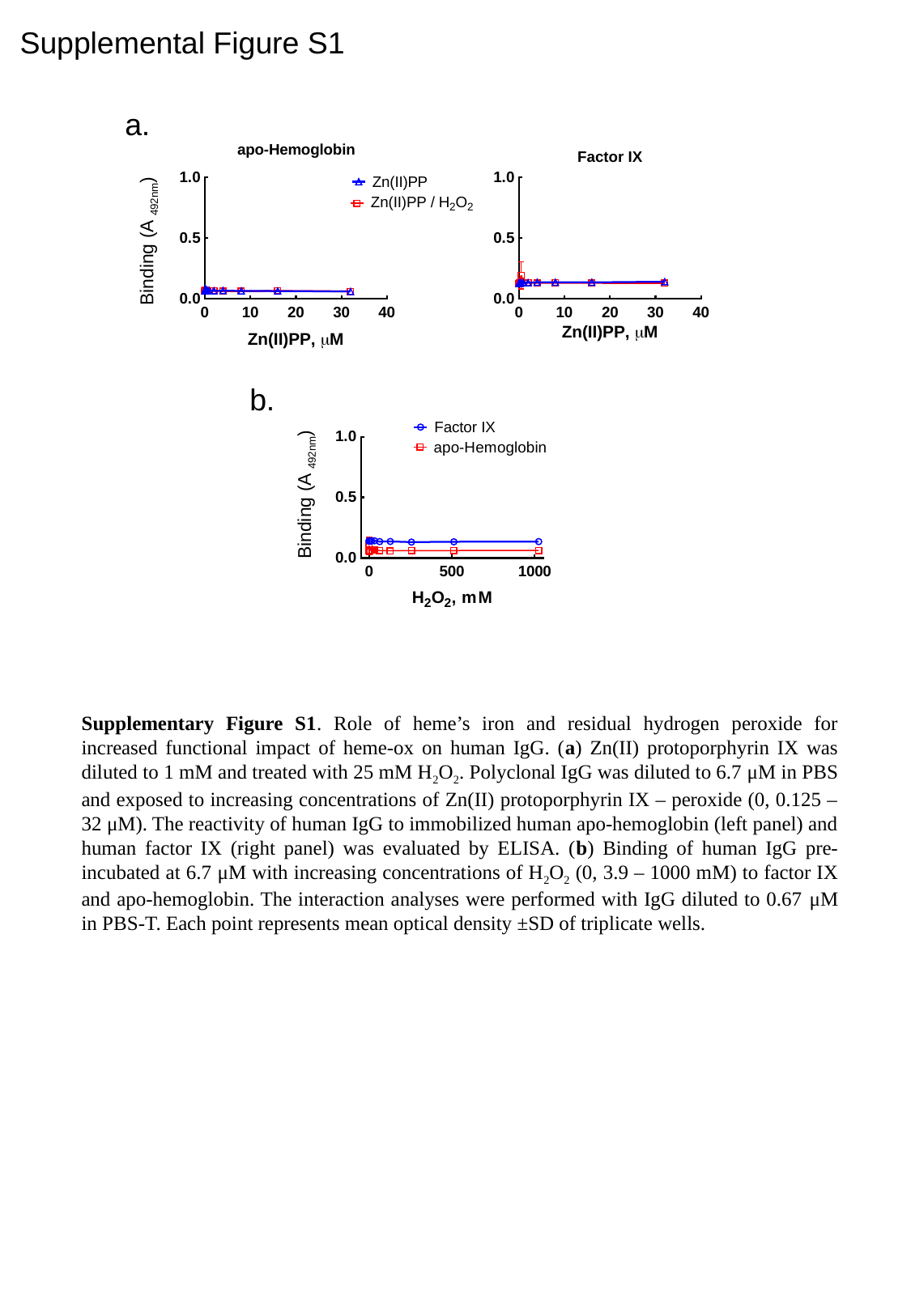

Supplemental Figure S1
a.
Binding (A 492nm)
b.
Binding (A 492nm)
Supplementary Figure S1. Role of heme’s iron and residual hydrogen peroxide for increased functional impact of heme-ox on human IgG. (a) Zn(II) protoporphyrin IX was diluted to 1 mM and treated with 25 mM H2O2. Polyclonal IgG was diluted to 6.7 μM in PBS and exposed to increasing concentrations of Zn(II) protoporphyrin IX – peroxide (0, 0.125 – 32 μM). The reactivity of human IgG to immobilized human apo-hemoglobin (left panel) and human factor IX (right panel) was evaluated by ELISA. (b) Binding of human IgG pre-incubated at 6.7 μM with increasing concentrations of H2O2 (0, 3.9 – 1000 mM) to factor IX and apo-hemoglobin. The interaction analyses were performed with IgG diluted to 0.67 μM in PBS-T. Each point represents mean optical density ±SD of triplicate wells.
